# *Trichomonas vaginalis* follow-up and persistence in Colombian women

**DOI:** 10.1038/s41598-021-02135-z

**Published:** 2021-11-19

**Authors:** Lauren Hernández-Buelvas, Milena Camargo, Ricardo Sánchez, Manuel Elkin Patarroyo, Manuel Alfonso Patarroyo

**Affiliations:** 1 Molecular Biology and Immunology Department, Fundación Instituto de Inmunología de Colombia (FIDIC), Carrera 50#26-20, 111321 Bogotá, DC Colombia; 2 MSc in Microbiology Programme, Universidad Nacional de Colombia, 111321 Bogotá, Colombia; 3 Animal Science Faculty, Universidad de Ciencias Aplicadas y Ambientales (U.D.C.A), 111166 Bogotá, Colombia; 4 Faculty of Medicine, Universidad Nacional de Colombia, 111321 Bogotá, Colombia; 5 Health Sciences Division, Main Campus, Universidad Santo Tomás, 110231 Bogotá, Colombia

**Keywords:** Parasitology, Risk factors

## Abstract

*Trichomonas vaginalis* (TV), the most common non-viral sexually-transmitted infection is considered a neglected infection and its epidemiology is not well known. This study determined TV-infection dynamics in a retrospective cohort of Colombian women and evaluated associations between risk factors and TV-outcome. TV was identified by PCR. Cox proportional risk models were used for evaluating the relationship between TV-outcome (infection, clearance and persistence) and risk factors (sexually-transmitted infections and sociodemographic characteristics). Two hundred and sixty-four women were included in the study; 26.1% had TV at the start of the study, 40.9% suffered at least one episode of infection and 13.0% suffered more than one episode of TV during the study. Women suffering HPV had a greater risk of TV-infection (aHR 1.59), high viral-load (> 10^2^) for HPV-16 being related to a greater risk of persistent parasite infection; a high viral load (> 10^2^) for HPV-18 and -33 was related to a lower probability of TV-clearance. Ethnicity (afrodescendent/indigenous people: aHR 5.11) and having had more than two sexual partners (aHR 1.94) were related to greater risk of infection, contrasting with women having a background of abortions and lower probability of having TV (aHR 0.50). Women aged 35- to 49-years-old (aHR 2.08), increased years of sexual activity (aHR 1.10), multiple sexual partners (aHR 8.86) and multiparous women (aHR 3.85) led to a greater probability of persistence. Women whose cervical findings worsened had a 9.99 greater probability of TV-persistence. TV distribution was high in the study population; its coexistence with HPV and other risk factors influenced parasite infection dynamics. The results suggested that routine TV detection should be considered regarding populations at risk of infection.

## Introduction

*Trichomonas vaginalis* (TV) is a protozoan parasite; it is considered the cause of highly prevalent sexually-transmitted infections (STI)^1^. Around 156 million cases are reported annually^2^. Chronic infections (usually following an asymptomatic clinical course) have serious consequences, such as chronic pelvic pain syndrome (CPPS)^1,3,4^.

A clinically relevant characteristics of *T. vaginalis* is that it has been shown to increase the risk of acquiring other STIs including *C. trachomatis* which has been associated with parasitic infection^5^; Previous studies have indicated that *C. trachomatis*-related concurrent infection is a predictive factor for acquiring *T. vaginalis*^6,7^. The strongest association has been described with *M. hominis*^8^; the symbiotic relationship between bacteria and the parasite involves the bacteria optimising *T. vaginalis* adenosine triphosphate (ATP) production, thereby facilitating its replication within the parasite^9,10^.

The relationship between *T. vaginalis* and human papillomavirus (HPV) has been demonstrated recently^11^. Studies have suggested that the internalisation and inflammation resulting from parasite infection produces changes in epithelial integrity, promoting the appearance of micro-abrasions facilitating HPV entry to the cervical epithelium’s differentiated base layers^12,13^. This is why parasite infection is considered a key risk factor regarding HPV persistence^12–14^. Understanding the factors involved in HPV persistence is thus relevant as it has been clearly demonstrated that the virus’ permanence in an organism is a key factor in cervical cancer (CC) development^15,16^.

The parasite has been little studied worldwide in spite of TV infection’s significance, thereby contributing to the lack of attention being paid to it by public health entities^17^; data about its distribution and the natural history of TV-infection clinical course has been little explored^18,19^. This could be leading to further problems, such as underreporting diagnoses and a lack of suitable control strategies^20^. The panorama is similar for Colombia; few studies have evaluated trichomoniasis epidemiology, meaning that the parasite’s infection dynamics have not been comprehensively evaluated.

This study was aimed at determining TV infection, clearance and persistence dynamics in a cohort of women from three Colombian cities (Chaparral, Girardot and Bogotá). In addition, the longitudinal association between risk factors was evaluated (i.e. sexual behaviour, HPV detection, *M. hominis* and *C. trachomatis*). Such information is extremely relevant as the burden of trichomoniasis in Colombia remains unknown due to the lack of TV-related epidemiological surveillance.

## Results

### STI sociodemographic characteristics and distribution

This study involved 264 women; their mean age was 41.8 years-old (SD: 10.9). TV was detected in 83.3% (5/6) of the women who claimed afro or indigenous descent. Table 1 describes the other variables. Parasite infection was detected in 26.1% (64/264) of the women at the start of the study; cumulative prevalence was 67.0% (177/264) during the two-year follow-up. It was found that 40.9% (108/264) of the participants had had at least one episode of infection and 13.0% (23/177) had suffered more than one episode of TV. Supplementary Fig. S1 describes other STI frequency at the start of study.

**Table 1:** Sociodemographic characteristics and risk factors regarding the women included (n = 264) in the retrospective component.

	*T. vaginalis*				
	Negative		Positive		
	Mean (SD)				
Age in years	42.0 (11)		41.2 (22.4)		
Years of active sex life	23.1 (10.8)		10.7 (10.3)		

	n	%	n	%	*p*
**City**^**a**^					
Bogotá	62	32.6	17	25.4	0.368
Other	128	67.4	50	74.6	
**Ethnicity**^**b**^					
Other	1	0.5	5	7.5	0.005
Mestizo	189	99.5	62	92.5	
**Marital status**^**c**^					
Status 1	18	9.5	7	10.5	0.817
Status 2	172	90.5	60	89.5	
**Age on first sexual relationship**					
≤ 18 years	112	58.9	38	56.7	0.350
> 18 years	78	41.1	29	43.3	
**Amount of sexual partners**					
1	91	48.9	26	39.4	0.402
2 or 3	77	41.4	33	50.0	
> 3	18	9.7	7	10.6	
**Amount of children**					
0–1	70	37.6	26	39.4	0.208
≥ 2	116	62.4	40	60.6	
**Family planning method**^**d**^					
No method	61	34.0	30	48.4	0.252
Hormonal	25	14.0	6	9.7	
Other	93	52	26	41.9	
**Abortions**					
No	80	56.3	25	48.1	0.306
Yes	62	43.7	27	51.9	
**Active STI**					
No	20	10.3	12	17.4	0.119
Yes	175	89.7	57	82.6	
**Smoker**					
Yes	48	25.4	13	19.4	0.720
No	141	74.6	54	80.6	
**A history of STI**					
No	138	70.4	53	77.9	0.717
Yes	58	29.6	15	22.1	

*STI* sexually-transmitted infection.

^a^City: The ‘other’ category included Girardot and Chaparral.

^b^Ethnicity: The ‘other’ category included Afro-Colombian and indigenous people.

^c^The ‘marital status 1’ category included single and separated women and widows; ‘marital status 2’ included married women and women living with a partner/free union.

^d^Family planning method ‘other’ included barrier methods and surgery.

No information regarding baseline colposcopy findings could be found for 10 of the women; colposcopy findings were negative for the remaining 81.1% (n = 206) whilst some type of abnormality was identified in 18.9% of them (n = 48), low-grade-squamous intraepithelial lesions (LSIL) occurring with the greatest frequency (Supplementary Fig. S2a). Changes in colposcopy findings were determined according to TV-outcome (Supplementary Fig. S2b).

### TV infection and risk factors

Infection rate was 3.4 per 100 people/month; survival functions showed that half the target population had acquired TV-infection 18.5 months after the start of the study (Supplementary Fig. S3a). Cox univariate and multivariate models were used for evaluating the associations between TV-infection and risk factors (Supplementary Tables S1–S5). Bivariate (Supplementary Table S1) and multivariate model results (Table 2) showed that afrodescendent/indigenous women had a greater probability (aHR 5.11) of acquiring parasite infection than mestizas; women having had more than 2 sexual partners (aHR 1.94) and/or active STI (aHR 1.81) were other infection-related factors. Women having a background of abortions (aHR 0.50) (Table 2) had a lower probability of acquiring TV.

**Table 2:** Hazard ratio adjusted for modelling the relationship between risk factors and TV-outcome.

Variable	*T. vaginalis*								
	Infection			Clearance			Persistence		
	aHR^a^	95% CI	*p*	aHR^a^	95% CI	*p*	aHR^a^	95% CI	*p*
**Ethnicity**									
Mestizo	Reference			Reference			Reference		
Other^b^	**5.11**	**2.02–12.93**	**0.001**	1.07	0.59–1.91	0.814	0.24	0.03–1.98	0.189
**Age in years**									
17–34	Reference			Reference			Reference		
35–49	1.39	0.63–3.06	0.403	1.08	0.44–2.65	0.852	**2.08**	**1.12–3.88**	**0.020**
> 49	2.50	0.81–7.71	0.109	0.66	0.24–1.82	0.434	2.01	0.93–4.36	0.075
**Marital status**^**c**^									
Status 1	Reference			Reference			Reference		
Status 2	0.54	0.28–1.02	0.061	1.31	0.63–2.69	0.459	0.66	0.37–1.16	0.155
Years of active sex life	0.96	0.93–1.00	0.094	1.00	0.96–1.03	0.970	**1.10**	**1.02–2.19**	**0.009**
**Amount of sexual partners**									
1	Reference			Reference			Reference		
2–3	**1.94**	**1.07–3.51**	**0.028**	0.74	0.45–1.21	0.241	0.31	0.08–6.23	0.313
> 3	1.89	0.79–4.55	0.151	1.36	0.74–2.49	0.318	**8.86**	**5.13–12.33**	**0.001**
**Pregnancies**									
0–1	Reference			Reference			Reference		
≥ 2	1.63	0.93–2.85	0.083	1.57	0.74–3.31	0.231	**3.85**	**2.16–8.81**	**0.027**
**Contraceptive method**									
No method	Reference			Reference			Reference		
Hormonal	1.08	0.44–2.65	0.860	1.90	0.42–8.62	0.330	3.21	0.40–9.57	0.269
Other^d^	1.22	0.73–2.03	0.430	0.98	0.65–1.47	0.927	1.63	0.86.5.56	0.148
**Abortions**									
No	Reference			Reference			Reference		
Yes	**0.50**	**0.27–0.94**	**0.032**	0.91	0.41–2.01	0.825	0.96	0.48–1.90	0.908
**Active STI**^**e**^									
No	Reference			Reference			Reference		
Yes	**1.81**	**1.10–2.98**	**0.019**	1.50	0.42–2.97	0.243	1.03	0.98–1.08	0.211

*HR* hazard ratio, *95% CI* 95% confidence interval, *STI* sexually transmitted infection.

Values in bold indicate *p* ≤ 0.05.

^a^Hazard ratio adjusted for ethnicity, age, marital status, years of active sex life, amount of sexual partners, pregnancies, contraceptive method used, abortions and active STI.

^b^Ethnicity: the other category includes Afro-descendants and indigenous people.

^**c**^Marital status: Status 1 included single, separated and widowed women; Status 2 included married women and those in common-law relationships.

^d^Contraceptive method: others includes barrier methods and surgery.

^e^Active STI included the detection of CH, HPV and MH.

**Figure 1:**
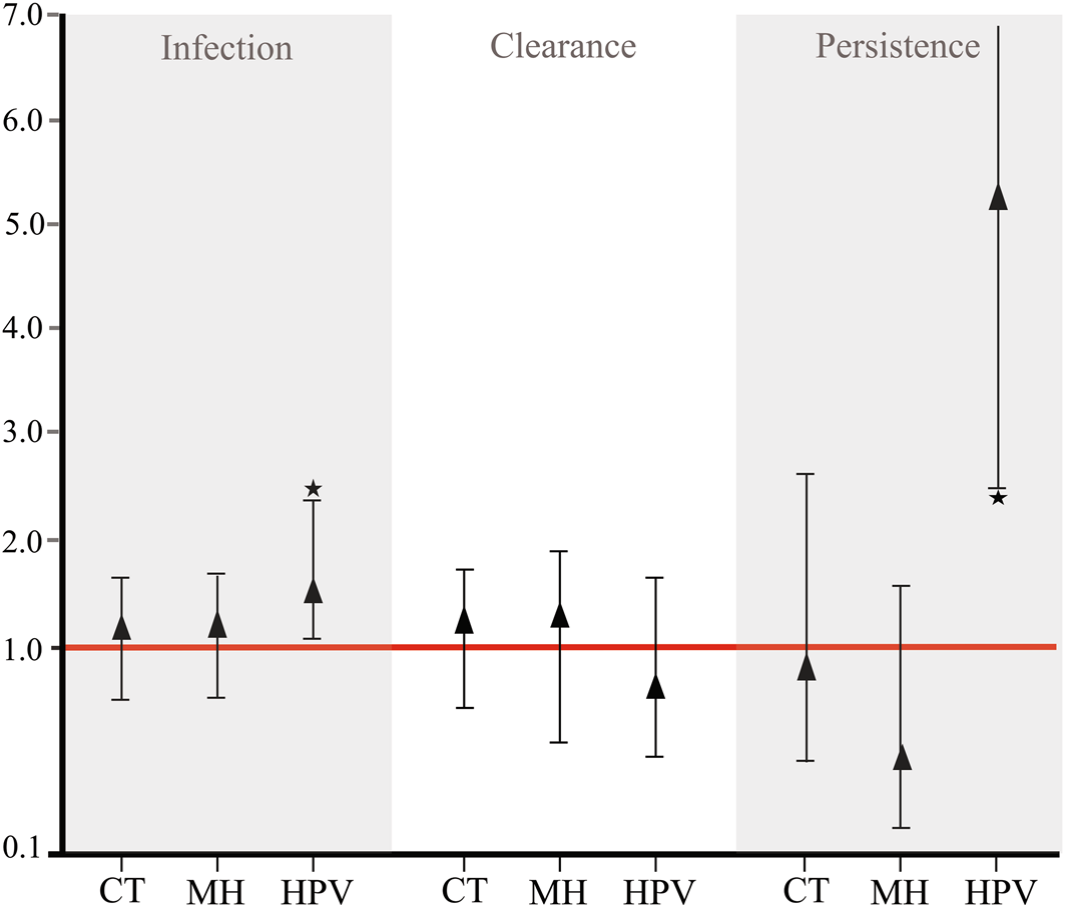
Adjusted hazard ratio for modelling the relationship between CT, MH and HPV and TV-outcome (infection, clearance and persistence). The reference group consisted of those women who were CT-, MH- or HPV-free.

**Figure 2:**
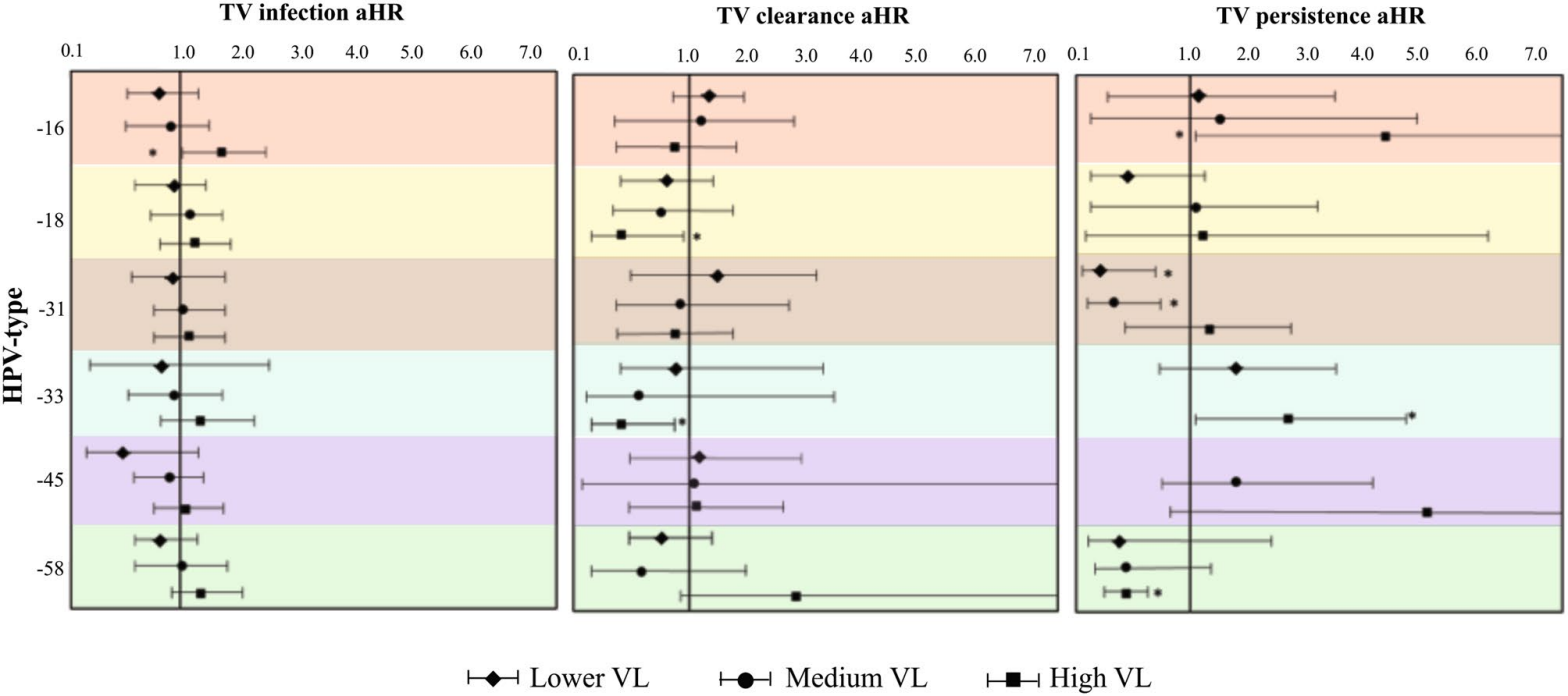
Risk ratios for modelling associations between TV infection, clearance and persistence and type-specific viral load. VL considered the amount of *HMBS* gene copies.

Regarding active STI, TV-infection was only associated with HPV where women having viral infection had a greater probability of parasite infection (aHR 1.59) (Fig. 1). No significant associations were observed for the six aforementioned hrHPV types (considering only presence or absence) with TV-infection (Supplementary Tables S2, S3). Nevertheless, new associations were revealed when evaluating the influence of the amount of hrHPV copies on parasite infection; this showed that women having a high HPV-16 VL (> 10^2^ copies) had a greater probability of having TV-infection (aHR 1.49) (Fig. 2, Supplementary Table S5). By contrast, having a low HPV-45 VL (less than 10^0^ copies) led to a lower probability (HR = 0.30) of TV-infection (Supplementary Table S4); such association was not significant after adjusting the multivariate model (Fig. 2).

### TV clearance and risk factors

The clearance rate was 15.0 per 100 people/month for TV-infected women when the study began (n = 69). Around 50.0% of the women cleared infection in a 6-month period (Supplementary Fig. S3b). HPV infection was associated with parasite clearance, showing that HPV-33 (aHR 0.49) (mainly in infections having high VL (> 10^2^ copies)) had a lower probability of TV-clearance (aHR 0.46); the same association was found with high HPV-18 VL (aHR 0.49) (Fig. 2). Women having worsened cervical lesion findings (identified by colposcopy) had a lower probability of parasite clearance (aHR 0.26) (Table 3).

**Table 3:** Adjusted hazard ratio for modelling the relationship between change in colposcopy results and TV-outcome.

Change in colposcopy	*T. vaginalis*								
	Infection			Clearance			Persistence		
	aHR^a^	95% CI	*p*	aHR^a^	95% CI	*p*	aHR^a^	95% CI	*p*
Alike	Reference			Reference			Reference		
Improved	0.85	0.59–1.22	0.388	1.21	0.41–3.51	0.725	**0.23**	**0.06–0.87**	**0.032**
Worsened	0.93	0.53–1.60	0.799	**0.26**	**0.08–0.77**	**0.016**	**9.99**	**3.16–31.59**	**0.001**

*HR* hazard ratio, *95% CI* 95% confidence interval, *STI* sexually transmitted infection.

Values in bold indicate *p* ≤ 0.05.

^a^Hazard ratio adjusted for ethnicity, age, marital status, years of active sex life, amount of sexual partners, pregnancies, contraceptive method used, abortions and active STI.

### TV persistence and risk factors

It was found that 5.6% of TV-infection did not become eliminated during the study period; persistence rate was 22.7 per 100 people/month. Regarding associated factors, there was a greater probability of persistence in women aged 35- to 49-years-old (aHR 2.08), those having increased years of sexual activity (aHR 1.10) having had more than three sexual partners (aHR 8.86) and multiparity (aHR 3.85) (Table 2).

Women having a high HPV-33 had a greater probability of TV-persistence (aHR 2.87) as did those having high HPV-16 VL (aHR 4.35). By contrast, low VL for HPV-31 and high VL for HPV-58 had a lower probability (Fig. 2).

Regarding colposcopy changes, women whose colposcopy findings improved had a lower probability (aHR 0.23) of TV-persistence whilst findings that worsened had 9.99 times more probability of TV-persistence (Table 3).

### HPV relationship with TV

Parasite influence on HPV infection outcome was evaluated in the study population as hrHPV permanence in an organism predisposes a host to CC development and TV infection can promote such viral permanence. The results showed that women suffering parasite infection had a greater probability (aHR 2.61; 1.07–6.37 95% CI; *p* = 0.034) of acquiring HPV infection and persistence (aHR 1.18; 1.00–1.75 95% CI; *p* = 0.050). No significant associations with viral elimination were observed (aHR 0.93; 0.44–2.44 95% CI; *p* = 0.931). Such results suggested that such coexistence works in two ways enabling both virus and parasite to perpetuate their infection in a targeted host.

## Discussion

A quarter of the women who began the study were infected by TV; such prevalence was greater than that reported from the USA^1^, Senegal^3^ and Brazil^21^, but similar to that found in other studies involving a heterogeneous population^6,22,23^. Discrepancies regarding frequency could have been due to target population composition, sample source and the parasite detection technique used^3,21,23^. As in many other countries, TV-infection is not targeted by any surveillance programme in Colombia, nor is it obligatory to report it; this could lead to underdiagnosis, underreporting and limitations regarding access to treatment^19,24^.

A significant association was found between lesion progression and parasite persistence (Table 3). TV-infections are characterised by damage to epithelial cell integrity, inducing inflammation and marked neutrophil infiltration^19^. This promotes a microenvironment facilitating the infection and persistence of other STIs, such as HPV^25^. The cervix’s base layers can become exposed and easily colonised by the virus as parasite infection leads to progressive damage of the cervical epithelium; parasite-virus coexistence induces pathogen persistence, thereby promoting the appearance and advance of cervical epithelium lesions^3,13,26^.

This study has reported a greater probability of HPV-infected women acquiring TV and its persistence (Fig. 2); such association is supported by other studies which have reported that women having HPV are at a greater risk of concurrent parasite infections^13,27^. Our results showed that HPV-16, -31 and -33 were mainly related to the clinical course of TV infection, an association previously described for TV and HPV-16^13^. This viral type is considered to be one of those having the greatest oncogenic potential as reflected by its high frequency in the population. Studies have highlighted the relationship between an increase in viral copies (facilitating their integration into host genome) and the grade of an intraepithelial lesion^28^; viral infections (mainly those having high VL) could become boosted by TV-derived pathogenesis.

Previous studies have reported HPV-18, -31 and -33 (HPV-31 and -33 being phylogenetically close to HPV-16) as being related to acquiring bacterial STIs (i.e. bacterial vaginosis)^23,29^; our study revealed associations between these hrHPV types and parasite infection (Fig. 2). Parasite-virus coexistence (sharing the same transmission route) favours pathogen colonisation and survival in a targeted host^30^. It has been suggested that parasite substrate production (altering cell membranes and facilitating viral infection), changes in local microbiota (by modulating the cervical microenvironment, thereby promoting the exploitation of vaginal substrates and improving pathogen virulence) and cysteine protease production that can degrade antibodies) can all alter a host’s immune response, as such biological interactions could modulate parasite-virus coexistence^29,31^.

In addition to HPV, such parasitosis has been related to non-viral STIs such as CT, MH and, recently, *M. girerdii*. Some of these bacteria colonise the parasite, becoming located intracellularly as a mechanism for evading a host’s immune response^26^; nevertheless, our results did not reveal significant parasite-bacteria associations (Fig. 1). Some studies which have evaluated TV and CT load have stressed the relationship between increased load and the appearance of specific symptoms; this has suggested their prognostic relevance regarding such infections’ clinical course^6^. However, information regarding CT and MH was not available for this study, thereby constituting a limitation and could have contributed towards the lack of perceived associations with these STI.

Regarding HPV, a higher VL was related to a greater risk of TV infection/persistence and a lower probability of parasite clearance (Fig. 2). The importance of the amount of HPV copies has been highlighted since their increase has been seen to be related to the appearance of cervical lesions (a dose–response relationship)^16^ and a greater probability of STI transmission^32^. An increase in the amount of viral copies leads to an increase in the amount of infected cells and viral DNA integration, meaning that many cellular targets are required for successful HPV propagation^33^. TV infection-derived pathogenesis (inflammation of the cervical mucosa and loss of cell integrity) predisposes the cervical epithelium and promotes HPV infection and persistence in a targeted host.

Our results showed that host factors (age and increased years of active sexual life) promoted TV-persistence (Table 2); such association can be partly explained by the effect of cervical hormones, as it has been described that constant oestrogen and progesterone levels (characteristic of older women or those leading an active sexual life) are linked to TV-infection susceptibility. These hormones promote cervical epithelium cell proliferation in a host, creating a suitable microenvironment for parasite colonisation and perpetuating its life-cycle^34^.

Other factors regarding host behaviour (i.e. multiparity and the amount of sexual partners) were seen to be related to parasite persistence (Table 2); these factors could alter vaginal microbiome equilibrium dynamics, changing homeostasis to a state of dysbiosis. Such imbalances regarding the local microbial environment could modulate a host’s immune response, thereby creating states of chronic inflammation which promote persistent parasite infection^26^. Having many sexual partners is a risk habit contributing to pathogen dispersion via sexual route^26,30^.

This study has reported a lower probability of infection in women having a history of abortions (Table 2); inverse relationships between other STIs and women having a history of abortions have been described^35^. Inflammatory processes and parasite infection-derived cytokine production could lead to premature births; the parasite’s vertical transmission leads to neonatal respiratory complications. It is currently known that TV causes limited vaginal infection which does not colonise the placenta and may not compromise foetal viability^15,36^; however, other studies have reported an association between TV-infection and serious adverse reproductive results^3^, meaning that further studies should be carried out for understanding the parasite’s role regarding reproductive health.

Ethnicity (afro/indigenous descent) was a factor related to a greater probability of TV-infection (Table 2); a relationship between high parasite infection prevalence and being an afrodescendent have been described previously^17^. Host characteristics (i.e. genetic background, HLA allele polymorphism), risky sexual behaviour and limitations regarding access to health services could explain the relationship between TV-infection and particular ethnic groups^1,17^. It should be stated that very few non-mestizo women were included in this study; although studies have reported that Afrodescendent women are more often infected with *T. vaginalis*, the socioeconomic and behavioural configuration could be totally different for Colombia, meaning that future studies should consider including a greater amount of Afro-Colombian women in them.

This study has indicated significant TV infection outcome-related associations. Sample size was a limitation of this study; future studies should thus include a larger population (mainly regarding particular ethnic groups) and should be carried out in such a way as to support the results obtained to date.

It is known that most TV-infections are asymptomatic; this means that such infections’ epidemiology and true burden remain unknown in terms of public health; TV is currently considered a neglected parasitosis, especially regarding limited socioeconomic settings. Understanding the factors related to TV dynamics and the influence of risk factors is relevant to research in this field and poses fresh challenges regarding the approach to and introduction of successful strategies for improving the female population’s quality of life.

## Methods

### Study design and ethical considerations

This study dealt with part of a bidirectional cohort; the prospective component (carried out between April 2007 and March 2010) was aimed at determining the natural history of infection by human papillomavirus (HPV) and *Chlamydia trachomatis* (CT). It involved obtaining cervical scrape samples from 17- to 69-year-old women from three Colombian cities: Chaparral in Colombia’s Tolima department, Girardot in Cundinamarca and Bogotá^15,37^. The retrospective component (this one) involved a group of women for whom at least four samples were available (a base-line and three follow-up samples) which had been taken 6 months (± 3 months) apart between visits (Supplementary Fig. S4).

All the women included in the study had signed an informed consent form authorising sample use for both the prospective and retrospective studies; an informed consent was obtained from a parent and/or legal guardian for younger than 18 year-old participants. The women had filled in a questionnaire for compiling sociodemographic information and data concerning their reproductive history and sexual behaviour. All the protocols had been evaluated and approved by the participating hospitals’ Ethics Committees^15,37^: Hospital de Engativá Nivel II in Bogotá (CEHE-009), Hospital San Juan Bautista in Chaparral in the Tolima department (10-CE-0197) and Nuevo Hospital San Rafael in Girardot in the Cundinamarca department (CEHG-024). All the methods were performed in accordance with the Helsinki declaration and Colombian Ministry of Health and Social Protection guidelines.

### Colposcopy and molecular detection of STI

Information about cervical architecture (by colposcopy) and the detection of two STI at all follow-up points (HPV and CT) was available for this study*.* Colposcopy had been carried out in line with technical guidelines established for Colombia, being performed and read by each participating hospital’s healthcare service; results were classified according to the Bethesda system. HPV and CT amplification conditions have been described previously^15,37^. Briefly, quantitative polymerase chain reaction (qPCR) was used for HPV detection and quantification; the primers and probes targeted *E1*, *E6*, *E7* genes for high-risk HPV (hrHPV) types HPV-16, -18, -31, -33, -33, -45 and -58. The human hydroxymethylbilane synthase (*HMBS*) gene was quantified as normaliser, along with the viral types^15^; conventional PCR (cPCR) was used for CT detection using two sets of primers targeting cryptic plasmid ORF2^37^.

TV and *Mycoplasma hominis* (MH) DNA was detected by cPCR in the retrospective component. Two sets of primers (Tvk3/7 and BTU9/2) were used for identifying TV DNA^36^; the RNAH1/2 set of primers was used for detecting MH DNA (Supplementary Table S6)^38^. Supplementary Table S7 describes the thermocycling conditions. The retrospective detection of TV in samples meant that the women who provided them (i.e. who were positive at the time) could not be treated with antiparasitic drugs.

### Statistical analysis

Measures of central tendency and dispersion were used for describing the quantitative variables; the categorical variables were expressed in terms of percentages, frequencies and cumulative prevalence (defined as the percentage of women infected by TV over a set period of time). Chi-squared or Fisher’s tests were used for evaluating differences regarding percentages.

TV-infection was defined as parasite DNA being detected at any moment during follow-up (2 years), clearance was defined as infection elimination (after having had a previous positive result for TV) and persistence as the non-elimination of parasite infection during the time the study lasted (Supplementary Fig. S5a).

The amount of hrHPV copies were grouped into viral load (VL) categories according to percentage distribution: negative or no infection, low VL (less than 10^0^ HPV copies), medium VL (10^0^ to 10^2^ HPV copies) and high VL (> 10^2^ HPV copies)^15^.

The baseline results and progression as the study advanced were considered when evaluating the prediction of cervical findings identified by colposcopy. These were categorised as ‘alike’ when initial colposcopy findings were similar to those on other occasions, ‘improved’ when some grade of lesion had been identified and improvement regarding an abnormality was observed during follow-up and ‘worsened’ when initial colposcopy report stated normal or low-grade lesion, yet the abnormality had advanced during follow-up (Supplementary Fig. S5b).

Kaplan–Meier survival analysis was used for estimating the probability of TV outcomes occurring throughout follow-up. Cox regression models were used for establishing the relationship between TV-outcome and CT, MH and HPV and risk factors. An additional model was run for establishing HPV-outcome (infection, clearance and persistence) association with TV (with or without parasite infection). All models were adjusted for the following covariables: smoker, ethnicity, age on first sexual relation, amount of sexual partners, family planning method, amount of abortions and contracting other STIs. The proportional hazards assumption was checked using statistical tests and graphical diagnostics based on scaled Schoenfeld residuals. STATA14 software was used for all two-tailed statistical tests (0.05 significance).

## Supplementary Information

Supplementary Information.

## Data Availability

The datasets produced and/or analysed during this study are available from the corresponding author on reasonable request.
