# Supplementary material for: Trichomonas vaginalis follow-up and persistence in Colombian women
Source: Sci Rep. 2021 Nov 19;11:22597. doi: 10.1038/s41598-021-02135-z (PMC8604905; doi:10.1038/s41598-021-02135-z)
Supplement: Supplementary file 1 — Supplementary Information. [file 41598_2021_2135_MOESM1_ESM.docx]

**Infection, clearance and persistence dynamics of *Trichomonas vaginalis* in a cohort of Colombian Women**

Lauren Hernández-Buelvas ^1,2†^, Milena Camargo ^1,3†^, Ricardo Sánchez ^4^, Manuel Elkin Patarroyo ^1,4,5^, Manuel Alfonso Patarroyo ^1,4,5*^

^1^ Molecular Biology and Immunology Department, Fundación Instituto de Inmunología de Colombia (FIDIC), Bogotá D.C. 111321, Colombia.

^2^ MSc Programme in Microbiology, Universidad Nacional de Colombia, Bogotá D.C. 111321, Colombia.

^3^ Animal Science Faculty, Universidad de Ciencias Aplicadas y Ambientales (U.D.C.A), Bogotá D.C. 111166, Colombia

^4^ Faculty of Medicine, Universidad Nacional de Colombia, Bogotá D.C. 111321, Colombia.

^5^ Health Sciences Division, Main Campus, Universidad Santo Tomás, Bogotá D.C. 110231, Colombia.

^†^ These authors contributed equally to this work as co-authors

^*^**Corresponding author:** Manuel Alfonso Patarroyo (MAP)

e-mail: [mapatarr.fidic@gmail.com](mailto:mapatarr.fidic@gmail.com)

Postal address: Carrera 50#26-20, Bogotá D.C. 111321, Colombia

Telephone number: +57-1-3244672 ext 141

**Supplementary Figure S1.** Baseline distribution of T. vaginalis (TV), M. hominis (MH), C. trachomatis (CT) and HPV.


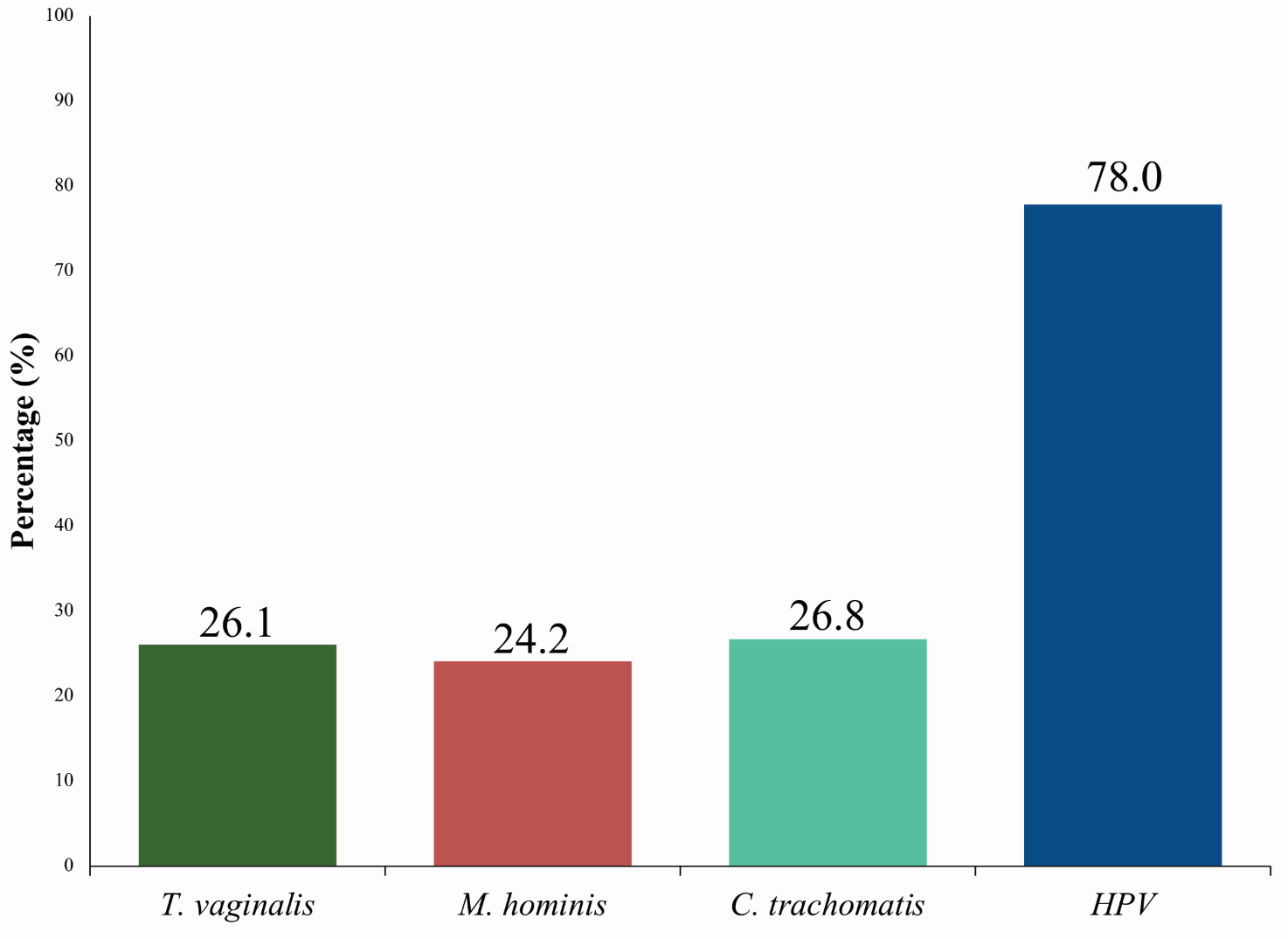


**Supplementary Figure S2.** Distribution of colposcopy findings and changes. (**a**) Distribution of colposcopy findings regarding follow-up. (**b**) Change in colposcopy results regarding TV outcome.


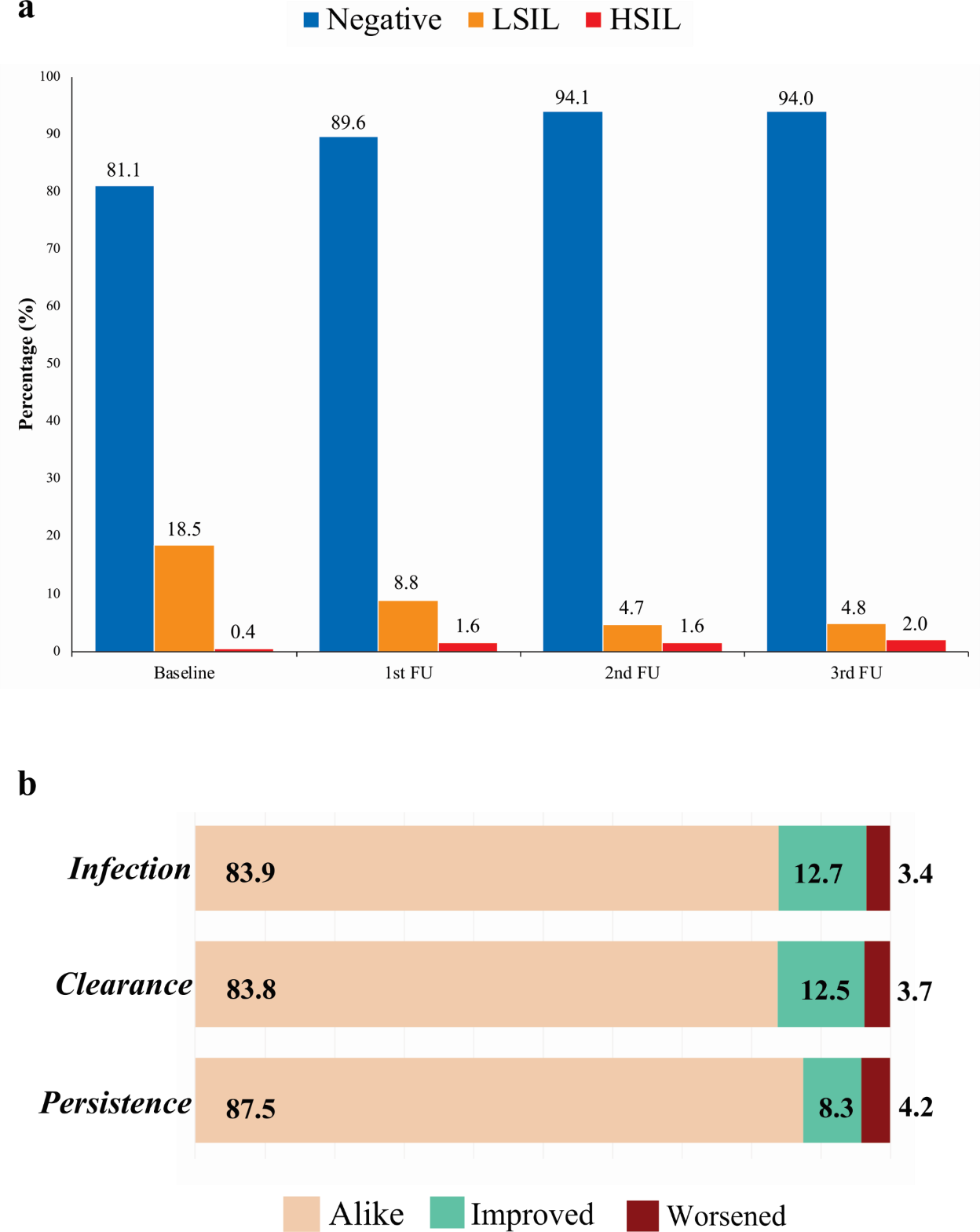


Abbreviations; LSIL: low-grade squamous intraepithelial lesion; HSIL: high-grade squamous intraepithelial lesion; 1st FU: first follow-up; 2nd FU: second follow-up; 3rd FU: third follow-up.

**Supplementary Figure S3.** Kaplan Meier curves regarding TV outcome. (**a)** Probability of infection. (**b)** clearance probability.


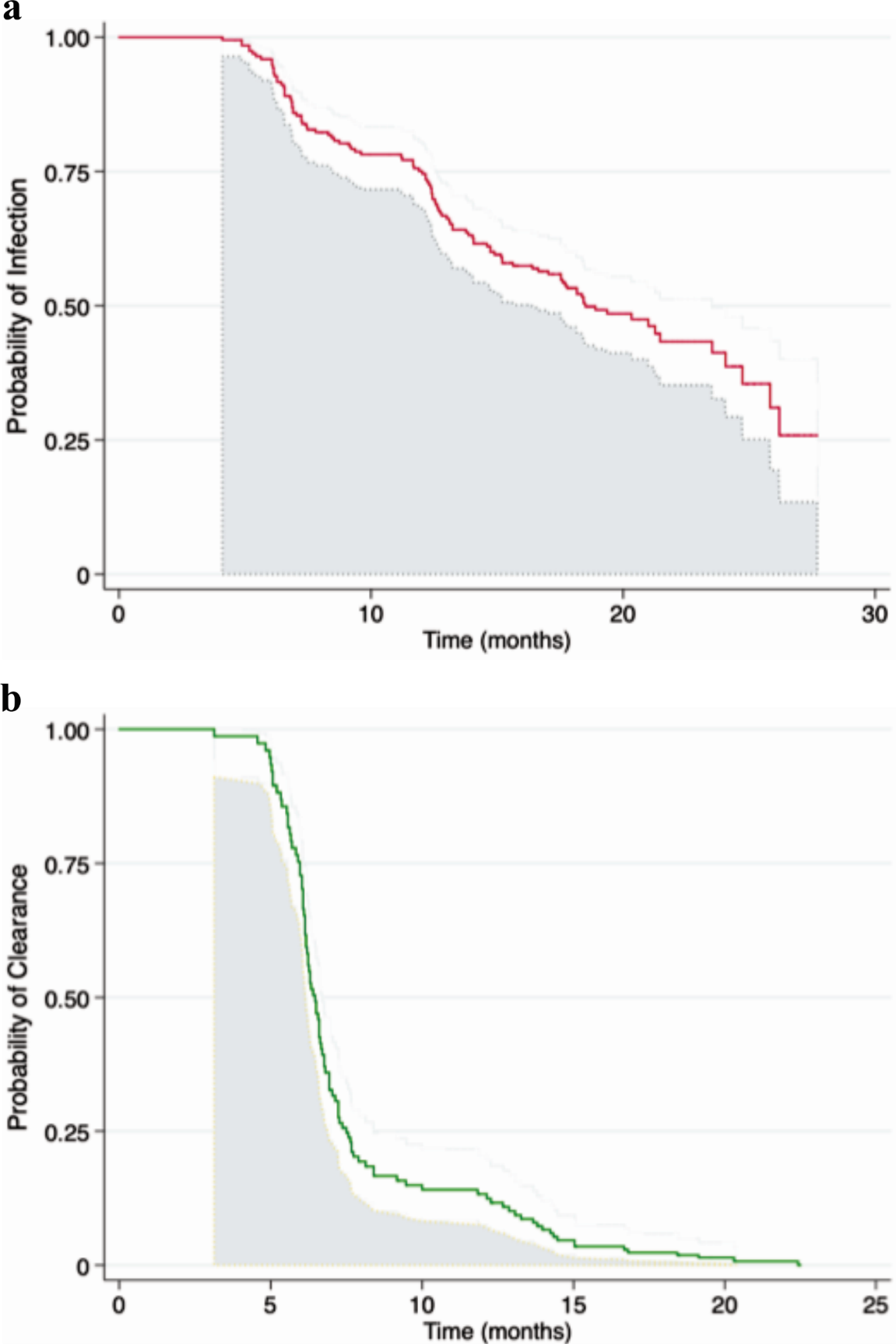


**Supplementary Table S1.** Bivariate hazard ratio for modelling the relationship between risk factors and TV outcome (infection, clearance and persistence).

| Variable | *T. vaginalis* | | | | | | | | | |
| --- | --- | --- | --- | --- | --- | --- | --- | --- | --- | --- |
|  | **Infection** | | | **Clearance** | | | **Persistence** | | | |
|  | **HR** | **95%CI** | ***p*** | **HR** | **95%CI** | ***p*** | **HR** | **95%CI** | | ***p*** |
| Ethnicity |  | | |  | | |  | | | |
| Mestiza | Reference | |  | Reference | |  | Reference | | |  |
| Other ^a^ | **10.40** | **6.48-16.7** | **0.001** | 0.95 | 0.59-1.54 | 0.852 | 0.83 | 0.32-2.16 | | 0.715 |
| Age in years |  |  |  |  | | |  | | | |
| 17-34 | Reference | |  | Reference | |  | Reference | | |  |
| 35-49 | 0.73 | 0.43-1.21 | 0.231 | 1.13 | 0.67-1.89 | 0.637 | 1.30 | 0.51-3.33 | | 0.571 |
| >49 | 0.79 | 0.49-1.27 | 0.337 | 0.88 | 0.56-1.39 | 0.600 | 1.12 | 0.44-2.84 | | 0.799 |
| Marital status ^b^ |  |  |  |  | | |  | | | |
| Status 1 | Reference | |  | Reference | |  | Reference | | |  |
| Status 2 | 0.61 | 0.35-1.07 | 0.090 | 1.21 | 0.96-1.52 | 0.103 | **1.66** | | **1.20-2.29** | **0.002** |
| Years of active sex life | 0.99 | 0.97-1.01 | 0.546 | 1.10 | 0.83-1.46 | 0.478 | 1.00 | | 0.97-1.03 | 0.970 |
| Amount of sexual partners |  |  |  |  | | |  | | | |
| 1 | Reference | |  | Reference | |  | Reference | | |  |
| 2-3 | **1.64** | **1.09-2.49** | **0.018** | 0.86 | 0.65-1.14 | 0.313 | 0.68 | | 0.42-1.10 | 0.121 |
| > 3 | 1.57 | 0.80-3.08 | 0.183 | 1.20 | 0.79-1.81 | 0.386 | **2.38** | | **1.34-4.25** | **0.003** |
| Amount of pregnancies |  |  |  |  | | |  | | | |
| 0-1 | Reference | |  | Reference | |  | Reference | | |  |
| ≥ 2 | 1.03 | 0.69-1.54 | 0.851 | 1.13 | 0.86-1.49 | 0.356 | 1.04 | | 0.57-1.89 | 0.893 |
| Contraceptive method |  |  |  |  | | |  | | | |
| No method | Reference | |  | Reference | |  | Reference | | |  |
| Hormonal | 0.93 | 0.49-1.79 | 0.847 | 1.29 | 0.68-2.46 | 0.421 | 1.23 | 0.61-2.46 | | 0.556 |
| Other ^c^ | 1.17 | 0.76-1.79 | 0.463 | 1.03 | 0.78-1.37 | 0.786 | 1.30 | 0.75-2.25 | | 0.336 |
| Abortions |  |  |  |  | | |  | | | |
| No | Reference | |  | Reference | |  | Reference | | |  |
| Yes | 0.77 | 0.48-1.24 | 0.287 | 0.96 | 0.69-1.33 | 0.824 | 0.75 | | 0.42-1.33 | 0.330 |
| Active STI |  |  |  |  | | |  | | | |
| No | Reference | |  | Reference | |  | Reference | | |  |
| Yes | 2.09 | 0.86-5.08 | 0.101 | 1.12 | 0.77-1.61 | 0.543 | 0.98 | | 0.27-3.45 | 0.976 |

Values in bold indicate *p*≤0.05

**^a^** Ethnicity: the ‘other’ category included afro and indigenous descent

**^b^** Marital status: Status 1 included single, separated and widowed women; Status 2 included married women and common-law partners

^c^ Contraceptive method: ‘other’ included barrier methods and surgery

Abbreviations: HR, hazard ratio; 95%CI: 95% confidence interval; STI, sexually transmitted infection

**Supplementary Table S2.** Bivariate hazard ratio for modelling the relationship between three sexually transmitted infections and TV outcome (infection, clearance and persistence).

| Other STI | *T. vaginalis* | | | | | | | | | | | |
| --- | --- | --- | --- | --- | --- | --- | --- | --- | --- | --- | --- | --- |
|  | **Infection** | | | **Clearance** | | | | **Persistence** | | | | |
|  | **HR** | **95%CI** | ***p*** | **HR** | **95%CI** | ***p*** | | **HR** | | **95%CI** | ***p*** | |
| *C. trachomatis* |  |  |  |  |  |  |  | | |  |  |  |
| Negative | Reference | |  | Reference | |  | Reference | | | |  |  |
| Positive | 1.06 | 0.66-1.70 | 0.738 | 0.98 | 0.74-1.29 | 0.901 | 0.80 | | | 0.46-1.38 | 0.428 |  |
| *M. hominis* |  |  |  |  |  |  |  | | |  |  |  |
| Negative | Reference | |  | Reference | |  | Reference | | | |  |  |
| Positive | 1.09 | 0.66-1.79 | 0.729 | 1.15 | 0.83-1.60 | 0.391 | 0.91 | | | 0.52-1.58 | 0.747 |  |
| HPV |  |  |  |  |  |  |  | | |  |  |  |
| Negative | Reference | |  | Reference | |  | | Reference | | |  | |
| Positive | 1.62 | 0.86-3.05 | 0.131 | 0.94 | 0.68-1.30 | 0.721 | | 1.51 | | 0.75-3.05 | 0.241 | |
| HPV-16 |  |  |  |  | | | |  | | | | |
| Negative | Reference | |  | Reference | |  | | Reference | | |  | |
| Positive | 0.85 | 0.49-1.48 | 0.579 | 1.19 | 0.89-1.57 | 0.224 | | 1.06 | | 0.61-1.82 | 0.825 | |
| HPV-18 |  |  |  |  | | | |  | | | | |
| Negative | Reference | |  | Reference | |  | | Reference | | |  | |
| Positive | 1.00 | 0.64-1.56 | 0.981 | **0.74** | **0.55-0.98** | **0.037** | | 1.15 | 0.64-2.05 | | 0.627 | |
| HPV-31 |  |  |  |  | | | |  | | | | |
| Negative | Reference | |  | Reference | |  | | Reference | | |  | |
| Positive | 1.50 | 0.93-2.42 | 0.088 | 0.80 | 0.60-1.06 | 0.134 | | 1.44 | 0.81-2.58 | | 0.210 | |
| HPV-33 |  |  |  |  | | | |  | | | | |
| Negative | Reference | |  | Reference | |  | | Reference | | |  | |
| Positive | 1.32 | 0.70-2.49 | 0.388 | 0.70 | 0.41-1.20 | 0.202 | | **2.21** | **1.09-4.47** | | **0.027** | |
| HPV-45 |  |  |  |  | | | |  | | | | |
| Negative | Reference | |  | Reference | |  | | Reference | | |  | |
| Positive | 0.68 | 0.42-1.11 | 0.125 | 0.99 | 0.73-1.34 | 0.972 | | 1.41 | | 0.75-2.63 | 0.280 | |
| HPV-58 |  |  |  |  | | | |  | | | | |
| Negative | Reference | |  | Reference | |  | | Reference | | |  | |
| Positive | 0.97 | 0.62-1.52 | 0.915 | 1.03 | 0.78-1.38 | 0.791 | | 0.79 | 0.45-1.38 | | 0.420 | |

Values in bold indicate *p*≤0.05

**Supplementary Table S3.** Adjusted hazard ratio for modelling the relationship between three sexually transmitted infections and TV outcome (infection, clearance and persistence).

| Other STIs | *T. vaginalis* | | | | | | | | |
| --- | --- | --- | --- | --- | --- | --- | --- | --- | --- |
|  | **Infection** | | | **Clearance** | | | **Persistence** | | |
|  | **aHR^a^** | **95%CI** | ***p*** | **aHR^a^** | **95%CI** | ***p*** | **aHR^a^** | **95%CI** | ***p*** |
| *C. trachomatis* |  |  |  |  |  |  |  |  |  |
| Negative | Reference | |  | Reference | |  | Reference | |  |
| Positive | 1.04 | 0.80-1.35 | 0.769 | 1.10 | 0.75-1.61 | 0.608 | 0.98 | 0.25-2.82 | 0.985 |
| *M. hominis* |  |  |  |  |  |  |  |  |  |
| Negative | Reference | |  | Reference | |  | Reference | |  |
| Positive | 1.05 | 0.82-1.36 | 0.666 | 1.15 | 0.67-1.97 | 0.607 | 0.48 | 0.21.1.66 | 0.250 |
| HPV |  | | |  | | |  | | |
| Negative | Reference | |  | Reference | |  | Reference | |  |
| Positive | **1.59** | **1.08-2.35** | **0.017** | 0.85 | 0.47-1.56 | 0.617 | **5.34** | **2.64-8.58** | **0.001** |
| HPV-16 |  |  |  |  | | |  | | |
| Negative | Reference | |  | Reference | |  | Reference | |  |
| Positive | 1.02 | 0.77-1.34 | 0.874 | 1.15 | 0.79-1.67 | 0.460 | 1.07 | 0.38-2.98 | 0.884 |
| HPV-18 |  |  |  |  | | |  | | |
| Negative | Reference | |  | Reference | |  | Reference | |  |
| Positive | 1.10 | 0.86- 1.41 | 0.414 | 0.67 | 0.43-1.06 | 0.093 | 0.89 | 0.34-2.34 | 0.823 |
| HPV-31 |  |  |  |  | | |  | | |
| Negative | Reference | |  | Reference | |  | Reference | |  |
| Positive | 1.15 | 0.90-1.48 | 0.247 | 0.75 | 0.50-1.12 | 0.173 | 0.66 | 0.38-1.14 | 0.137 |
| HPV-33 |  |  |  |  | | |  | | |
| Negative | Reference | |  | Reference | |  | Reference | |  |
| Positive | 1.15 | 0.85- 1.55 | 0.348 | **0.49** | **0.23-0.91** | **0.026** | **4.22** | **1.36-8.53** | **0.001** |
| HPV-45 |  |  |  |  | | |  | | |
| Negative | Reference | |  | Reference | |  | Reference | |  |
| Positive | 0.99 | 0.78-1.27 | 0.979 | 0.95 | 0.59-1.53 | 0.845 | 1.73 | 0.34-8.71 | 0.502 |
| HPV-58 |  |  |  |  | | |  | | |
| Negative | Reference | |  | Reference | |  | Reference | |  |
| Positive | 1.03 | 0.82-1.30 | 0.748 | 0.96 | 0.60-1.53 | 0.867 | 0.42 | 0.21.1.82 | 0.120 |

Values in bold indicate *p*≤0.05

^a^ Adjusted hazard ratio for ethnicity, age, marital status, years of active sexual life, amount of sexual partners, pregnancies, contraceptive method and abortions

Abbreviations: aHR, adjusted hazard ratio; 95%CI: 95% confidence interval; STI, sexually transmitted infection

**Supplementary Table S4.** Bivariate hazard ratio for modelling the relationship between six hrHPV types’ viral load and TV outcome (infection, clearance and persistence).

| Viral load | | *T. vaginalis* | | | | | | | | |
| --- | --- | --- | --- | --- | --- | --- | --- | --- | --- | --- |
|  |  | **Infection** | | | **Clearance** | | | **Persistence** | | |
|  |  | **HR** | **95%CI** | ***p*** | **HR** | **95%CI** | ***p*** | **HR** | **95%CI** | ***p*** |
| HPV-16 VL | Negative | Reference | |  | Reference | |  | Reference | |  |
|  | Low | 0.62 | 2.89-1.37 | 0.244 | 1.31 | 093-1.83 | 0.115 | 0.80 | 0.41-1.57 | 0.534 |
|  | Medium | 0.40 | 1.35-1.20 | 0.105 | 1.15 | 0.59-2.23 | 0.678 | 1.34 | 0.48-3.71 | 0.569 |
|  | High | **1.86** | **1.05-4.06** | **0.035** | 0.96 | 0.58-1.60 | 0.905 | 1.23 | 0.57-2.63 | 0.590 |
| HPV-18 VL | Negative | Reference | |  | Reference | |  | Reference | |  |
|  | Low | 0.90 | 0.47-1.74 | 0.768 | 0.83 | 0.59-1.16 | 0.291 | 0.69 | 0.33-1.46 | 0.344 |
|  | Medium | 0.90 | 0.48-1.69 | 0.760 | 0.77 | 0.52-1.61 | 0.219 | 1.39 | 0.56-3.42 | 0.464 |
|  | High | 1.17 | 0.62-2.20 | 0.620 | **0.45** | **0.23-0.89** | **0.023** | 1.39 | 0.65-2.96 | 0.382 |
| HPV-31 VL | Negative | Reference | |  | Reference | |  | Reference | |  |
|  | Low | 1.22 | 0.57-2.64 | 0.598 | 0.80 | 0.36-1.77 | 0.591 | 2.40 | 0.80-7.18 | 0.115 |
|  | Medium | 1.43 | 0.74-2.74 | 0.276 | 0.91 | 0.61-1.37 | 0.681 | 1.00 | 0.49-2.04 | 0.981 |
|  | High | 1.59 | 0.91-2.78 | 0.101 | 0.80 | 0.54-1.18 | 0.270 | 1.71 | 0.73-4.03 | 0.213 |
| HPV-33 VL | Negative | Reference | |  | Reference | |  | Reference | |  |
|  | Low | 0.65 | 0.07-5.31 | 0.691 | 0.90 | 0.35-2.33 | 0.843 | .077 | 0.20-2.83 | 0.696 |
|  | Medium | 0.55 | 0.11-2.54 | 0.446 | 0.73 | 0.30-1.75 | 0.491 | **20.4** | **9.04-39.8** | **0.001** |
|  | High | 2.02 | 0.86-4.75 | 0.105 | 0.79 | .34-1.82 | 0.592 | **2.32** | **1.10-4.89** | **0.026** |
| HPV-45 VL | Negative | Reference | |  | Reference | |  | Reference | |  |
|  | Low | **0.30** | **0.10-0.87** | **0.028** | 1.08 | 0.75-1.57 | 0.652 | - | - | - |
|  | Medium | 0.75 | 0.3-1.56 | 0.444 | 0.96 | 0.59-1.55 | 0.887 | 1.17 | 0.44-3.07 | 0.747 |
|  | High | 0.74 | 0.36-1.52 | 0.415 | 1.07 | 0.58-1.95 | 0.819 | 1.07 | 0.49-2.33 | 0.865 |
| HPV-58 VL | Negative | Reference | |  | Reference | |  | Reference | |  |
|  | Low | 0.74 | 0.41-1.31 | 0.306 | 0.97 | 0-66-1.42 | 0.894 | 0.77 | 0.38-1.58 | 0.490 |
|  | Medium | 1.32 | 0.55-3.15 | 0.528 | 0.89 | 0.62-1.27 | 0.548 | 0.63 | 0.29-1.36 | 0.245 |
|  | High | 1.24 | 0.51-3.00 | 0.622 | 2.17 | 0.82-5.75 | 0.117 | 0.86 | 0.34-2.16 | 0.750 |

Values in bold indicate *p*≤0.05

**Supplementary Table S5.** Adjusted hazard ratio for modelling the relationship between six hrHPV types’ viral load and TV outcome (infection, clearance and persistence).

| Viral load | | *T. vaginalis* | | | | | | | | |
| --- | --- | --- | --- | --- | --- | --- | --- | --- | --- | --- |
|  |  | **Infection** | | | **Clearance** | | | **Persistence** | | |
|  |  | **aHR ^a^** | **95%CI** | ***p*** | **aHR** | **95%CI** | ***p*** | **aHR** | **95%CI** | ***p*** |
| HPV-16 VL | Negative | Reference | |  | Reference | |  | Reference | |  |
|  | Low | 0.79 | 0.49-1.27 | 0.283 | 1.30 | 0.85-1.99 | 0.213 | 1.10 | 0.37-3.24 | 0.862 |
|  | Medium | 0.81 | 0.46-1.42 | 0.468 | 1.15 | 0.45-2.92 | 0.758 | 1.82 | 0.19-5.01 | 0.222 |
|  | High | **1.49** | **1.01-2.22** | **0.049** | 0.86 | 0.42-1.79 | 0.701 | **4.35** | **2.01-7.71** | **0.028** |
| HPV-18 VL | Negative | Reference | |  | Reference | |  | Reference | |  |
|  | Low | 0.94 | 0.6-1.40 | 0.772 | 0.78 | 0.48-1.28 | 0.336 | 0.49 | 0.17-1.42 | 0.195 |
|  | Medium | 1.02 | 0.71-1.44 | 0.911 | 0.73 | 0.32-1.65 | 0.459 | 1.02 | 0.33-3.15 | 0.967 |
|  | High | 1.20 | 0.88-1.65 | 0.240 | **0.49** | **0.27-0.90** | **0.023** | 1.15 | 0.21-6.24 | 0.871 |
| HPV-31 VL | Negative | Reference | |  | Reference | |  | Reference | |  |
|  | Low | 0.95 | 0.59-1.52 | 0.854 | 1.23 | 0.50-2.99 | 0.646 | **0.17** | **0.04-0.73** | **0.001** |
|  | Medium | 1.09 | 0.78-1.52 | 0.579 | 0.86 | 0.41-1.80 | 0.704 | **0.42** | **0.23-0.98** | **0.017** |
|  | High | 1.13 | 0.85-1.51 | 0.391 | 0.82 | 0.43-1.54 | 0.534 | 1.28 | 0.57-2.91 | 0.541 |
| HPV-33 VL | Negative | Reference | |  | Reference | |  | Reference | |  |
|  | Low | 0.82 | 0.31-2.19 | 0.699 | 0.82 | 0.48-3.18 | 0.781 | 1.80 | 0.87-3.72 | 0.109 |
|  | Medium | 0.90 | 0.50-1.62 | 0.744 | 0.64 | 0.13-3.20 | 0.459 | - | - | - |
|  | High | 1.34 | 0.86-2.08 | 0.185 | **0.46** | **0.29-0.78** | **0.001** | **2.87** | **1.01-4.86** | **0.001** |
| HPV-45 VL | Negative | Reference | |  | Reference | |  | Reference | |  |
|  | Low | 0.58 | 0.28-1.23 | 0.158 | 1.07 | 0.50-2.99 | 0.646 | - | - | - |
|  | Medium | 0.92 | 0.65-1.32 | 0.683 | 1.09 | 0.37-3.17 | 0.863 | 1.81 | 0.89-3.97 | 0.994 |
|  | High | 1.03 | 0.73-1.45 | 0.852 | 1.08 | 0.46-2.55 | 0.849 | 5.02 | 0.92-9.26 | 0.061 |
| HPV-58 VL | Negative | Reference | |  | Reference | |  | Reference | |  |
|  | Low | 0.88 | 0.64-1.21 | 0.458 | 0.85 | 0.48-1.50 | 0.579 | 0.42 | 0.09-1.99 | 0.280 |
|  | Medium | 1.02 | 0.64-1.61 | 0.920 | 0.65 | 0.27-1.53 | 0.326 | 0.41 | 0.11-1.43 | 0.164 |
|  | High | 1.33 | 0.92-1.93 | 0.123 | 2.86 | 0.91-9.00 | 0.071 | **0.42** | **0.35-0.51** | **0.001** |

Values in bold indicate *p*≤0.05

^a^ Adjusted hazard ratio for ethnicity, age, marital status, years of active sexual life, amount of sexual partners, pregnancies, contraceptive method, abortions and active STI

Abbreviations: aHR, adjusted hazard ratio; 95%CI: 95% confidence interval; HPV

**
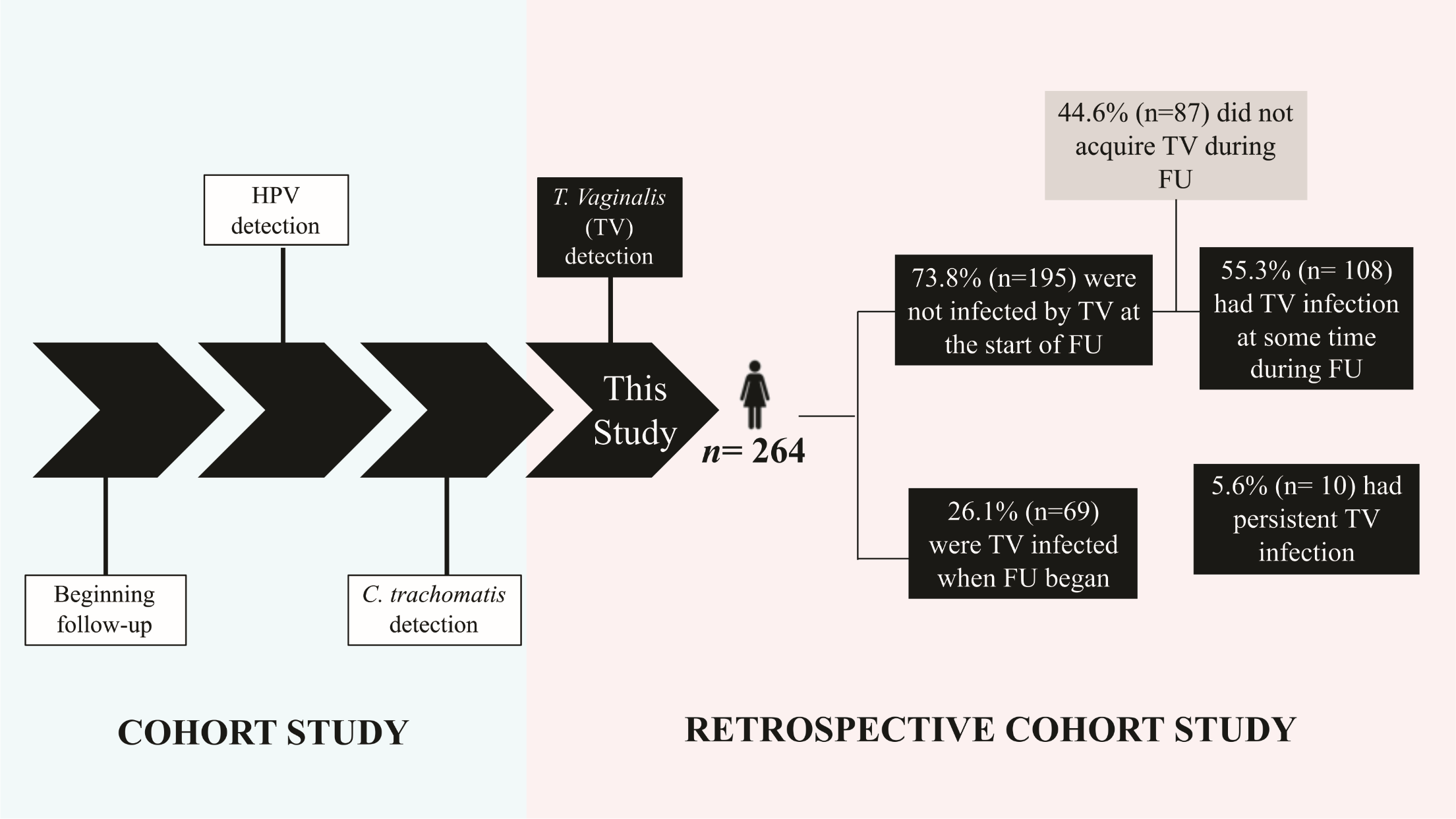
Supplementary Figure S4.** Flow diagram of study design.

**Supplementary Table S6.** Oligonucleotide primers used for STI detection.

| Primers | Primer direction | Primer sequence (5' - 3') | Size (bp) | Ref |
| --- | --- | --- | --- | --- |
| Tvk3/7 | Fwd | ATTGTCGAACATTGGTCTTACCCTC | 261 | [^1^](#_ENREF_1) |
|  | Rev | TCTGTGCCGTCTTCAAGTATGC |  |  |
| BTU9/2 | Fwd | CATTGATAACGAAGCTCTTTACGA | 112 |  |
|  | Rev | GCATGTTGTGCCGGACATAACCAT |  |  |
| RNAH1/2 | Fwd | CAATGGCTAATGGCCGGATACGC | 334 | [^2^](#_ENREF_2) |
|  | Rev | GGTACCGTCAGTCTGCAAT |  |  |

Fwd: forward; Rev: reverse*;* bp: base pairs; Ref: reference

**Supplementary Table S7.** Thermal cycler PCR conditions for each set of primers.

| Protocol | Programme | PCR | | |
| --- | --- | --- | --- | --- |
|  |  | **Temperature** | **Time** | **Cycles** |
| **TVK3/7** | Pre-incubation | 94°C | 5 minutes | 1 |
|  | Amplification | 94°C  60°C  72°C | 45 seconds | 40 |
|  | Final extension | 72°C | 7 minutes | 1 |
| **BTU9/2** | Pre-incubation | 94°C | 5 minutes |  |
|  | Amplification | 94°C  56°C  72°C | 45 seconds | 35 |
|  | Final extension | 72°C | 7 minutes | 1 |
| **RHAH1/2** | Pre-incubation | 94°C | 5 minutes | 1 |
|  | Amplification | 94°C  62°C  72°C | 45 seconds | 40 |
|  | Final extension | 72°C | 7 minutes | 1 |

**Supplementary Figure S5.** Schematic representation of events determined in the study. (**a)** Diagram regarding the definitions for infection, clearance and persistence outcomes in this study. (**b)** Diagram regarding how changes in colp

oscopy results were categorised.

**
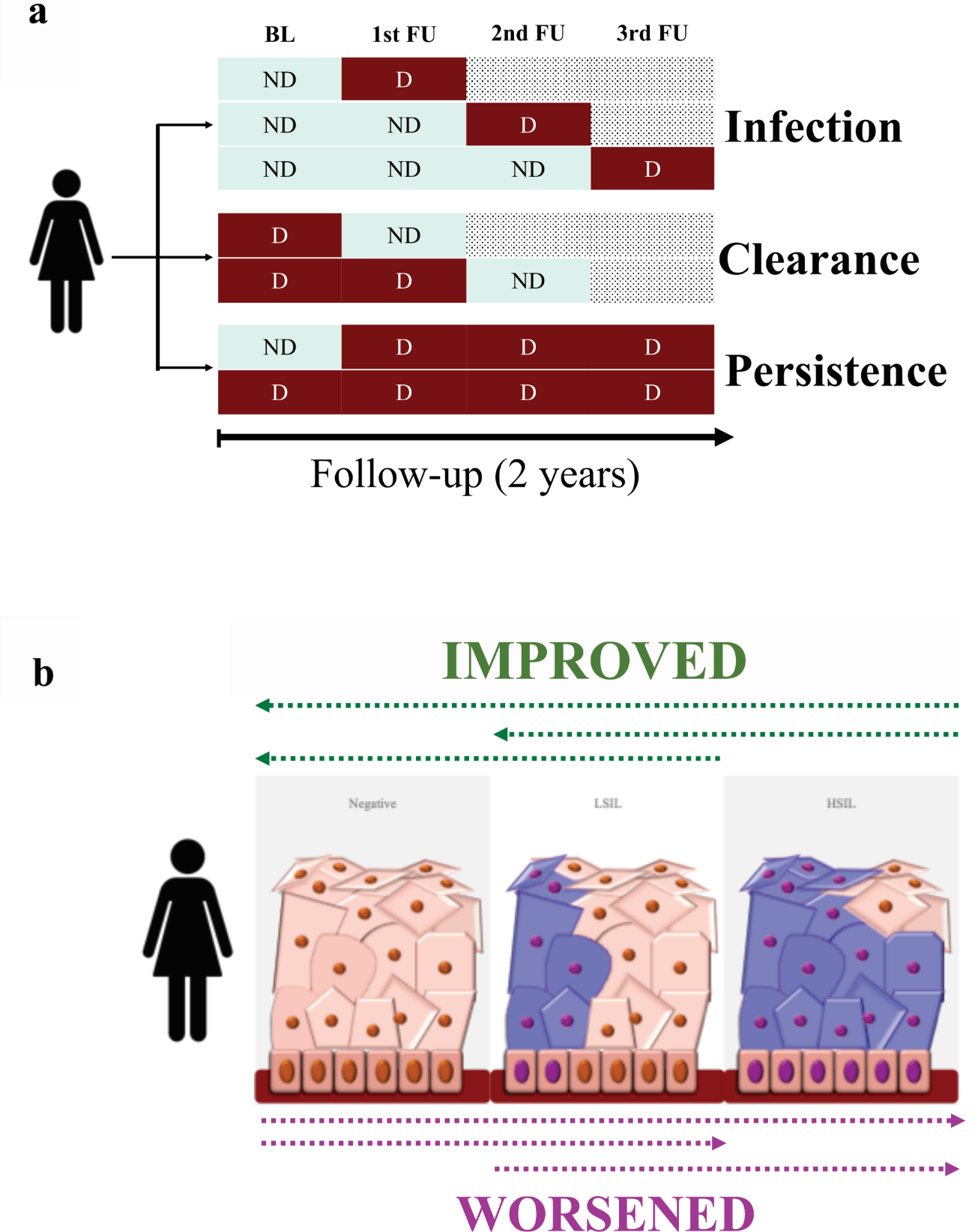
**

Abbreviations; 1st FU: first follow-up; 2nd FU: second follow-up; 3rd FU: third follow-up.

**References**

1 Goo, Y. K. *et al.* Prevalence of Trichomonas vaginalis in Women Visiting 2 Obstetrics and Gynecology Clinics in Daegu, South Korea. *Korean J Parasitol* **54**, 75-80 (2016).

2 Arjadi, R. & Patel, V. Q&A: Scaling up delivery of mental health treatments in low and middle income countries: interviews with Retha Arjadi and Vikram Patel. *BMC Med* **16**, 211 (2018).
